# Multiplexed Gene Engineering Based on dCas9 and gRNA-tRNA Array Encoded on Single Transcript

**DOI:** 10.3390/ijms24108535

**Published:** 2023-05-10

**Authors:** Chaoqian Jiang, Lishuang Geng, Jinpeng Wang, Yingjuan Liang, Xiaochen Guo, Chang Liu, Yunjing Zhao, Junxue Jin, Zhonghua Liu, Yanshuang Mu

**Affiliations:** 1 Key Laboratory of Animal Cellular and Genetic Engineering of Heilongjiang Province, Northeast Agricultural University, Harbin 150030, China; 2 College of Life Science, Northeast Agricultural University, Harbin 150030, China

**Keywords:** CRISPR/Cas9, multiplexed genome engineering, tRNA-gRNA array, triplex sequence

## Abstract

Simultaneously, multiplexed genome engineering and targeting multiple genomic loci are valuable to elucidating gene interactions and characterizing genetic networks that affect phenotypes. Here, we developed a general CRISPR-based platform to perform four functions and target multiple genome loci encoded in a single transcript. To establish multiple functions for multiple loci targets, we fused four RNA hairpins, MS2, PP7, com and boxB, to stem–loops of gRNA (guide RNA) scaffolds, separately. The RNA-hairpin-binding domains MCP, PCP, Com and λN22 were fused with different functional effectors. These paired combinations of cognate-RNA hairpins and RNA-binding proteins generated the simultaneous, independent regulation of multiple target genes. To ensure that all proteins and RNAs are expressed in one transcript, multiple gRNAs were constructed in a tandemly arrayed tRNA (transfer RNA)-gRNA architecture, and the triplex sequence was cloned between the protein-coding sequences and the tRNA-gRNA array. By leveraging this system, we illustrate the transcriptional activation, transcriptional repression, DNA methylation and DNA demethylation of endogenous targets using up to 16 individual CRISPR gRNAs delivered on a single transcript. This system provides a powerful platform to investigate synthetic biology questions and engineer complex-phenotype medical applications.

## 1. Introduction

Eukaryotic gene expression is a complex process controlled at multiple levels, including epigenetic, transcriptional and posttranscriptional regulation. High-throughput technologies have been used with multidimensional genomic datasets to study the cross-layer regulatory interplay [1,2]. Confirmation of the multidimensional network by using molecular biology methods is important to understand and elucidate the pathogenesis of cancer and other diseases [3,4,5]. The ability to manipulate genomic activities at different dimensions, such as gene expression and epigenetics, simultaneously is a prerequisite for identifying the comprehensive view of the gene’s state and further delineating the complex picture of disease development.

The clustered regularly interspaced short palindromic repeat (CRISPR)-Cas9 gene modulation system has been developed and widely used as a tool for multifunctional genome manipulation [6]. The deactivated Cas9 (dCas9), in conjunction with a guide RNA (gRNA), functions as a modular DNA-binding scaffold [7]. dCas9 has been subsequently fused with effector domains such as the VP64 transactivation domain, KRAB repression domain, Dnmt3a DNA methylation domain or Tet1 DNA demethylation domain, allowing manipulation of genome loci at multiple levels [7,8,9]. To perform a range of different editing events, the RNA aptamer strategy was used to form the multifunctional CRISPR system. In this system, aptamer–RNA-binding protein (ABP) fused with an effector domain can bind to their RNA hairpins with a specific secondary RNA structure. RNA hairpins, such as MS2, PP7, com and boxB, were fused to the stem–loop of gRNA scaffolds to recruit a functional domain to dCas9 via ABP interactions [10,11,12,13]. The specific interaction between the RNA hairpins and ABP enabled multifunctional CRISPR systems to work independently in the same cell [11,12]. This strategy has been used to recruit gene activation or repression effectors to target different genomic sites to perform dual-function systems [13], to recruit sets of fluorescent proteins to label multiplexed genomic sites [12] and to execute gene cytosine base editing (CBE), adenine base editing (ABE) and indel mutations on different targets to perform tri-function systems [14]. This system is uniquely suited for multiplex gene modulation or multiplex gene synergistic function.

To simultaneously target multiple distinct genomic loci, the multiplexed gRNAs need to be expressed and complexed with the Cas9 nuclease, which forms the CRISPR-Cas9 system. There are many methods to produce multiple functional gRNAs from a single transcript, such as using *Csy4* RNA ribonuclease [15] and ribozymes to process polycistronic gRNAs [16,17]. In these strategies, using a synthetic gene with a tandemly arrayed tRNA-gRNA architecture, each tRNA was cleaved near the 5′ and 3′ ends by the endogenous RNases RNase P and Z, respectively, and multiple gRNAs could be efficiently and precisely produced from a single precursor transcript in the nucleus. The tRNA-gRNA expression system has been successfully demonstrated in human cells, mice and plants [17,18,19]. This tRNA-gRNA strategy is an attractive and efficient method to facilitate the delivery method and vector capacity for the CRISPR system.

To construct the multifunctional CRISPR systems, the common strategy is to stack multiple independent expression cassettes on independent plasmids and deliver the constructs into cells by cotransfection or step-by-step transfection. Multiple plasmids lead to differences in the plasmid copy number and gene expression and make it difficult to achieve the desired effect. To overcome this limitation, the composition of multifunctional CRISPR systems should be simple. The triplex structure is a 110-nt RNA tertiary structure element derived from the 3′ end of the mouse noncoding-RNA-metastasis-associated lung adenocarcinoma transcript 1 (*Malat1*) and can stabilize transcripts lacking poly(A) tails through the formation of a tertiary structure element [20]. In previous studies, the triplex structure was used to enable the simultaneous expression of protein and gRNAs on the same transcript [21]. The triplex structure can also stabilize mRNAs after Cas12a-mediated RNA processing, enabling concomitant protein expression without affecting gene editing efficiency [22].

In this study, we developed a multifunctional CRISPR system for transcriptional activation, transcriptional repression, DNA methylation and DNA demethylation. By combining the tRNA-gRNA array and triplex structure, the CRISPR system can be encoded in a single transcript, simultaneously targeting 16 individual genome loci and performing four different biological functions. This multifunctional CRISPR system provides a platform to elucidate and control the gene network’s underlying complex cellular functions.

## 2. Results

### 2.1. Construction and Mechanism of the Multifunctional System Based on CRISPR/dCas9 and tRNA-gRNA Array

**Figure 1:**
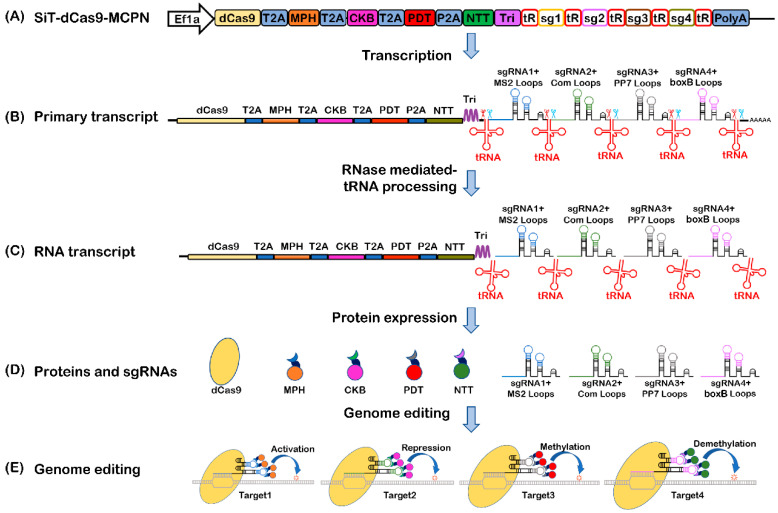
Schematic of the multiplexed genome engineering mechanism mediated by CRISPR/dCas9. (**A**) Schematic of the SiT-dCas9-MCPN vector. (**B**) Schematic of the primary RNA of the SiT-dCas9-MCPN vector. (**C**) The primary transcript of tRNA-gRNA array is cleaved by endogenous RNase P and RNase Z (labeled as scissors) to release mature gRNAs and tRNA. (**D**) The scheme proteins of dCas9, MPH, CKB, PDT and NTT proteins and the structure of gRNA scaffolds. (**E**) A scheme for CRISPR-dCas9-based gene modulation of activation, repression, methylation and demethylation. SiT-dCas9-MCPN, construction of a multiplexed genome engineering system; MPH, fusion of MCP with P65 and HSF1; CKB, fusion of Com with KRAB; PDT, fusion of PCP with Dnmt3a; NTT, fusion of N22p with TET1; T2A, 2A self-cleaving peptide derived from *Thosea asigna* virus; P2A, 2A self-cleaving peptide derived from porcine teschovirus-1; Tri, RNA triple helix structure.

To design the multifunctional system based on CRISPR/dCas9, gRNA scaffolds contain two copies of the MS2, PP7, com or boxB stem–loop motifs extended from the tetraloop and stem–loop, respectively. RNA-binding proteins (MCP, Com, PCP and λN22) were fused to functional protein domains, respectively. Fusion of MCP with P65 and HSF1 forms MCP-P65-HSF1 (MPH) as a transcriptional activation complex. Fusion of Com with the KRAB (Krüppel-associated box domain of Kox1) forms Com-KRAB (CKB) as a transcriptional repression complex. Fusion of PCP with Dnmt3a (DNA methyltransferase 3a) forms PCP-Dnmt3a (PDT) as a DNA methylation complex. Fusion of N22 with TET1 (ten-eleven 1 hydroxylase) forms N22-TET1 (NTT) as a DNA demethylation complex (Figure 1A). Each fusion protein adds one copy of the SV40 NLS and protein tag, HA, His, c-Myc or Flag, respectively. This multifunctional system was termed CRISPR-MCPN.

To enable the simultaneous expression of multiple proteins and gRNAs on the same transcript, we prepared a plasmid containing one mammalian promoter, an intron-less form of the human elongation factor 1a (EF1a), which permits efficient transgene expression in a variety of mammalian cells. All the proteins including dCas9, MPH, CKB, PDT and NTT flanked by 2A or P2A self-cleaving peptides were translated into a fusion protein, and the 2A self-cleaving peptide underwent self-cleavage to generate mature proteins. The RNA triple helix structure ensures protein transcript RNA stability and enhanced translational efficiency. tRNA^Gly^-gRNA arrays flanked by tRNAs were used to produce functional gRNAs. Thus, we successfully constructed a plasmid with fusion proteins, a 2A self-cleaving peptide, an RNA triple helix structure and a tRNA^Gly^-gRNA array for the simultaneous and efficient expression of multiple proteins and gRNAs in a single promoter (Figure 1A).

When the plasmid enters the cell nucleus, all the proteins, RNA triple helix structure and tRNA^Gly^-gRNA array were transcribed in one single transcript (Figure 1B). Then, tRNA^Gly^-gRNA arrays flanked by tRNAs were processed by endogenous RNase P and RNase Z, which cut the 5′ and 3′ ends, respectively, of pretRNAs to produce functional gRNAs (Figure 1C). The RNA triple helix structure stabilized protein transcripts lacking poly(A) tails through the formation of a tertiary structure element [20], which was added between proteins and the tRNA^Gly^-gRNA array and was a 110 nt structure derived from the 3′ end of the mouse noncoding-RNA-metastasis-associated lung adenocarcinoma transcript 1 (Malat1) (Figure 1C). This approach enables one single promoter to transcribe all proteins, RNA triple helix structure and tRNA^Gly^-gRNA precursors in one single transcript and release individual proteins and gRNAs, simultaneously (Figure 1D).

When the multifunctional system is used, the dCas9 and gRNA direct and bind specific target DNA sites. RNA-hairpin sequences (i.e., MS2, PP7, com and boxB) were fused to the gRNA scaffold to recruit specific functional regulators to dCas9 via aptamer–RNA-binding protein interactions. The specific interaction between the hairpins and RNA-binding proteins enabled four functions, including activation, repression, methylation and demethylation, to work independently in the same cell (Figure 1E).

### 2.2. Simultaneous Expression of Multiple Proteins and gRNA Scaffolds from a Single Pol II-Derived Transcript

**Figure 2:**
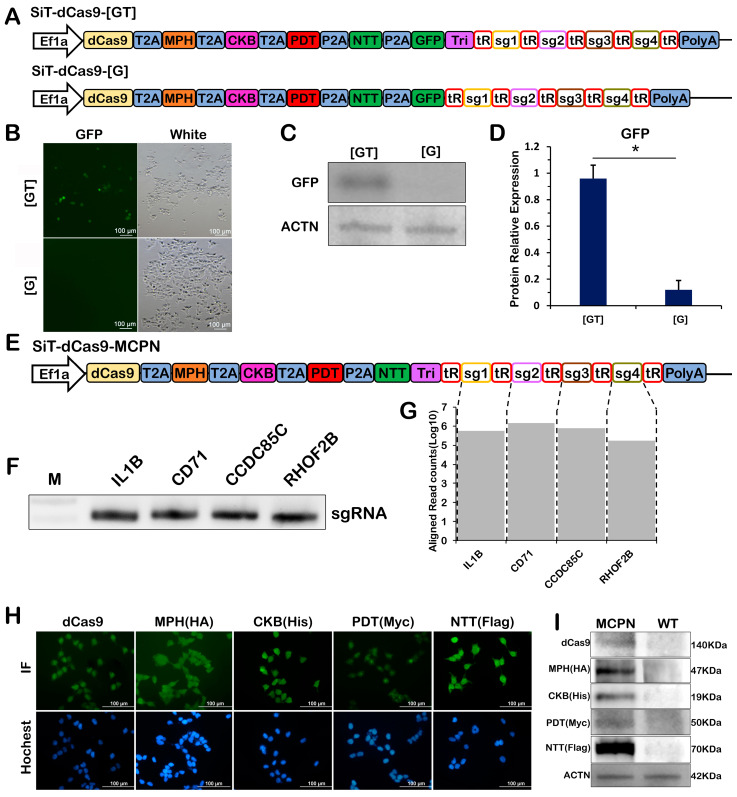
Simultaneous expression of proteins and gRNA scaffolds from a Pol II promoter. (**A**) Schematic of the SiT-dCas9-[GT] and SiT-dCas9-[G] vectors. (**B**) Representative EGFP fluorescent images after transfection of the SiT-dCas9-[GT] and SiT-dCas9-[G] plasmids. (**C**) Quantification of the expression of EGFP by Western blot. (**D**) Corresponding densitometric analyses of the protein bands of EGFP/β-actin. (**E**) Schematic of the SiT-dCas9-MCPN vectors. (**F**) Detection of mature gRNAs in cells expressing the SiT-dCas9-MCPN. (**G**) Quantification of mature gRNAs in cells expressing the SiT-dCas9-MCPN. (**H**) Representative photomicrographs of immunohistochemistry staining of dCas9, MPH, CKB, PDT and NTT (magnification, ×100). All these cells were selected by puro. (**I**) Western blots of dCas9, MPH, CKB, PDT and NTT. All these experiments were in HEK293T cells. MPH, fusion of MCP with P65 and HSF1; CKB, fusion of Com with KRAB; PDT, fusion of PCP with Dnmt3a; NTT, fusion of N22p with TET1; T2A, 2A self-cleaving peptide derived from Thosea asigna virus; P2A, 2A self-cleaving peptide derived from porcine teschovirus-1; Tri, RNA triple helix structure. Values represent the mean ± s.e.m., *n* = 3 independent experiments. * *p* < 0.05.

The simultaneous generation of multiple gRNA scaffolds and proteins from a single transcript is an important premise of this study. In this architecture, RNA cleavage by RNAases P and Z releases a functional gRNA but also removes the poly(A) tail from the upstream mRNA, which is known to result in an impaired translation of most eukaryotic mRNAs. To complement the RNAase P- and Z-mediated loss of the poly(A) tail, we cloned a 110 bp fragment derived from the 3′ end of the mouse MALAT1 locus [20] downstream of proteins and immediately upstream of the gRNA sequence flanked by tRNA. To confirm the simultaneous expression of multiple proteins, we added the EGFP coding sequence to the original five fusion proteins before the triplex and transfected the plasmid into HEK293T cells (Figure 2A). In cells transfected with four fusion proteins and EGFP transcripts containing tRNA-gRNA arrays, the expression of EGFP was observed compared to transcripts without the triplex (Figure 2B). The expression of EGFP was also confirmed by Western blotting (Figure 2C,D). In this MCPN system, we used a tRNA-gRNA array, which flanked gRNA by tRNA, to express multiple gRNAs. RNase P and Z recognized the tRNA, cleaved it, and released it, and mature gRNAs were detected by cRT-PCR and deep sequencing (Figure 2F,G). In addition, in cells transfected with five fusion proteins containing tRNA-gRNA arrays, the expression of dCas9 and four fusion proteins, including MPH, CKB, PDT and NTT, was detected by immunocytochemistry and Western blotting (Figure 2H,I). These results showed that each protein was expressed and existed independently. Taken together, these results suggest that the triplex sequence and tRNA-gRNA arrays enable concomitant protein expression and mature gRNAs in a single transcript.

### 2.3. Simultaneous Gene Activation, Gene Repression, DNA Methylation and DNA Demethylation with the CRISPR-MCPN System

**Figure 3:**
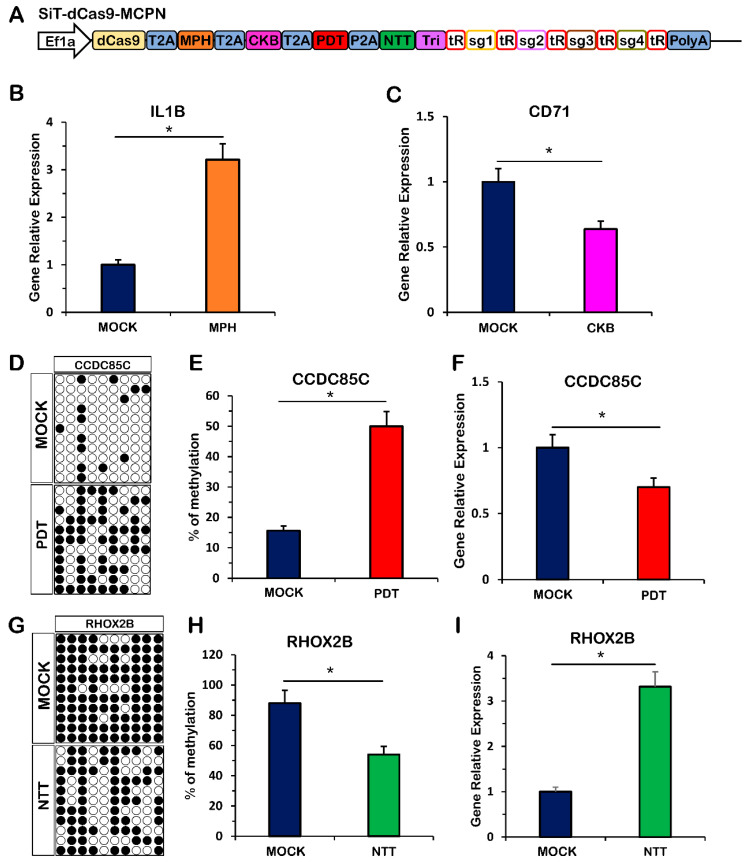
Simultaneous gene activation, gene repression, DNA methylation and DNA demethylation based on the dCas9 platform. (**A**) Schematic representation of the CRISPR-MCPN vector. (**B**) Quantification of relative RNA expression for the *IL1B* gene after multiplexed expression of 4 distinct gRNAs from the MCPN system. (**C**) Quantification of relative RNA expression for the *CD71* gene after multiplexed expression of 4 distinct gRNAs from the MCPN system. (**D**) Methylation levels of each individual CpG in the *CCDC85C* promoter region. (**E**) Methylation levels of each individual CpG in the *CCDC85C* promoter region. (**F**) Quantification of relative RNA expression for the *CCDC85C* gene after multiplexed expression of 4 distinct gRNAs from the MCPN system. (**G**) Methylation levels of each individual CpG in the *RHOFX2B* promoter region. (**H**) Methylation levels of each individual CpG in the *RHOFX2B* promoter region. (**I**) Quantification of relative RNA expression for the *RHOFX2B* gene after multiplexed expression of 4 distinct gRNAs from the MCPN system. MOCK, untransfected cells; MPH, fusion of MCP with P65 and HSF1; CKB, fusion of Com with KRAB; PDT, fusion of PCP with Dnmt3a; NTT, fusion of N22p with TET1; T2A, 2A self-cleaving peptide derived from Thosea asigna virus; P2A, 2A self-cleaving peptide derived from porcine teschovirus-1; Tri, RNA triple helix structure. Values represent the mean ± s.e.m., *n* = 3 independent experiments. * *p* < 0.05.

To evaluate the potential of the CRISPR-MCPN platform for multiplexed genome functions, we used a tRNA-gRNA array containing four distinct gRNA scaffolds, which contained two copies of the MS2, PP7, com or boxB, targeting four endogenous genomic loci (*IL1B*, *CD71*, *CCDC85C* and *RHOX2B*), and quantified the system efficiency (Figure 3A). These results showed that *IL1B* gene expression was activated by MPH (Figure 3B). *CD71* gene expression was repressed by CKB (Figure 3C). The CGC island of the *CCDC85C* gene promoter was methylated by PDT (Figure 3D,E), and *CCDC85C* expression was downregulated (Figure 3F). The CGC island of the *RHOX2B* gene promoter was demethylated by NTT (Figure 3G,H), and *RHOX2B* expression was upregulated (Figure 3I). Overall, these data indicate that the CRISPR-MCPN platform enables multigenome function for gene activation, gene repression, genome methylation and genome demethylation simultaneously.

### 2.4. Specific Interaction of the MS2, PP7, Com or λ N22 RNA Stem–Loop with Its RNA-Binding Protein

**Figure 4:**
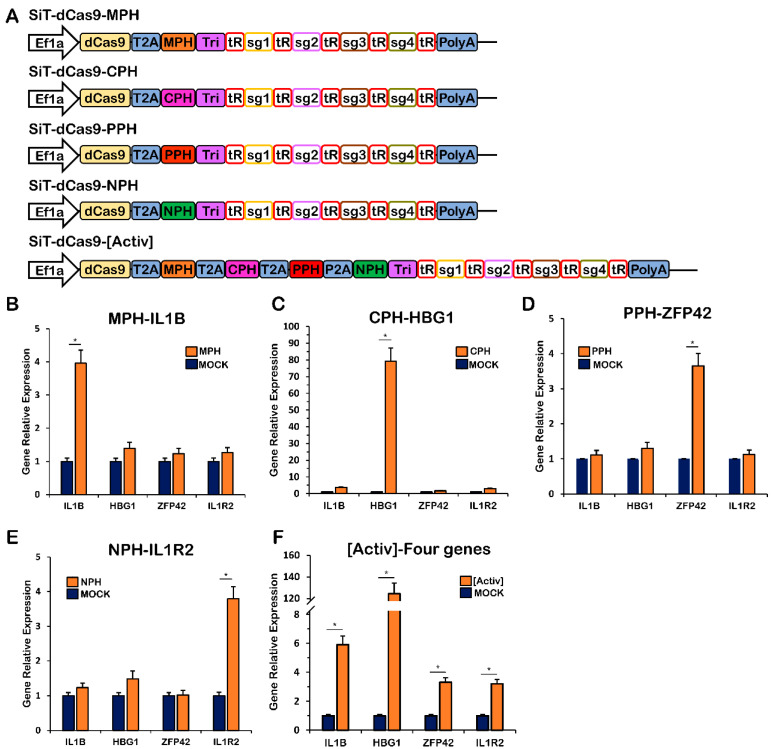
The specific interaction of MS2, PP7, com or λ N22 RNA stem–loops assembled into RNA scaffolds with their cognate-RNA-binding proteins. (**A**) Schematic representation of the CRISPR active vectors. (**B**) Quantification of the relative RNA expression of the *IL1B*, *HBG1*, *ZFP42* and *IL1R2* genes after the expression of the SiT-dCas9-MPH-targeted IL1B gene. (**C**) Quantification of the relative RNA expression of the *IL1B*, *HBG1*, *ZFP42* and *IL1R2* genes after the expression of the SiT-dCas9-CPH-targeted *HBG1* gene. (**D**) Quantification of the relative RNA expression of the *IL1B*, *HBG1*, *ZFP42* and *IL1R2* genes after the expression of the SiT-dCas9-PPH-targeted *ZFP42* gene. (**E**) Quantification of relative RNA expression of *IL1B*, *HBG1*, *ZFP42* and *IL1R2* genes after the expression of SiT-dCas9-NPH-targeted *IL1R2* gene. (**F**) Quantification of relative RNA expression of *IL1B*, *HBG1*, *ZFP42* and *IL1R2* genes after the expression of SiT-dCas9-[Activ]-targeted *IL1B*, *HBG1*, *ZFP42* and *IL1R2* genes. MPH, fusion of MCP with P65 and HSF1; CPH, fusion of Com with P65 and HSF1; PPH, fusion of PCP with P65 and HSF1; NPH, fusion of N22p with P65 and HSF1; T2A, 2A self-cleaving peptide derived from Thosea asigna virus; P2A, 2A self-cleaving peptide derived from porcine teschovirus-1; Tri, RNA triple helix structure. Values represent the mean ± s.e.m., *n* = 3 independent experiments. * *p* < 0.05.

To establish a broad spectral range for the multifunctional system, we fused MS2, PP7, com or λ N22 hairpins to the tetraloop and stem–loop of the gRNA scaffolds. The resulting gRNAs each recruited the RNA-hairpin-binding domains recognizing the cognate-RNA elements, which were fused with specific functional domains. To determine the specificity of crosstalk between RNA hairpins and cognate-binding proteins (e.g., MS2 RNA recruiting the MCP protein), we fused the RNA-hairpin-binding domains, including MCP, PCP, Com or λ N22, with HSF1 transcriptional activators as the transcriptional activation complex. Fusion of MCP with P65 and HSF1 forms MCP-P65-HSF1 (MPH), fusion of PCP with P65 and HSF1 forms PCP-P65-HSF1 (PPH), fusion of Com with P65 and HSF1 forms Com-P65-HSF1 (CPH), and fusion of λ N22 with P65 and HSF1 forms N22-P65-HSF1 (NPH). Then, we constructed the plasmid containing one of the four fused proteins and the tRNA-gRNA array to generate four RNA scaffolds, which each contained MS2, PP7, com or λ N22 RNA hairpins targeted to four separate genes (Figure 4A). We also constructed a plasmid containing all four fused proteins and the four tRNA-gRNA-array-targeted genes (Figure 4A). The *IL1B*, *HBG1*, *ZFP42* and *IL1R* genes were used as reporter genes to detect any weak cross-activation between RNA hairpins and RNA-hairpin-binding domains. The results showed that SiT-dCas9-MPH, SiT-dCas9-CPH, SiT-dCas9-PPH and SiT-dCas9-NPH accurately activated the target genes *IL1B*, *HBG1*, *ZFP42* and *IL1R*, respectively (Figure 4B–E). SiT-dCas9-[Activ] could activate all four genes (Figure 4F). These results suggested that no significant crosstalk was detected between mismatched pairs of RNA hairpin and binding proteins. The strong activation of reporter gene expression only with cognate-RNA hairpin and RNA-binding protein pairs demonstrates the potential for the simultaneous, independent regulation of multiple target genes.

### 2.5. Simultaneous Multiplexed Genome Editing with the CRISPR-MCPN System

**Figure 5:**
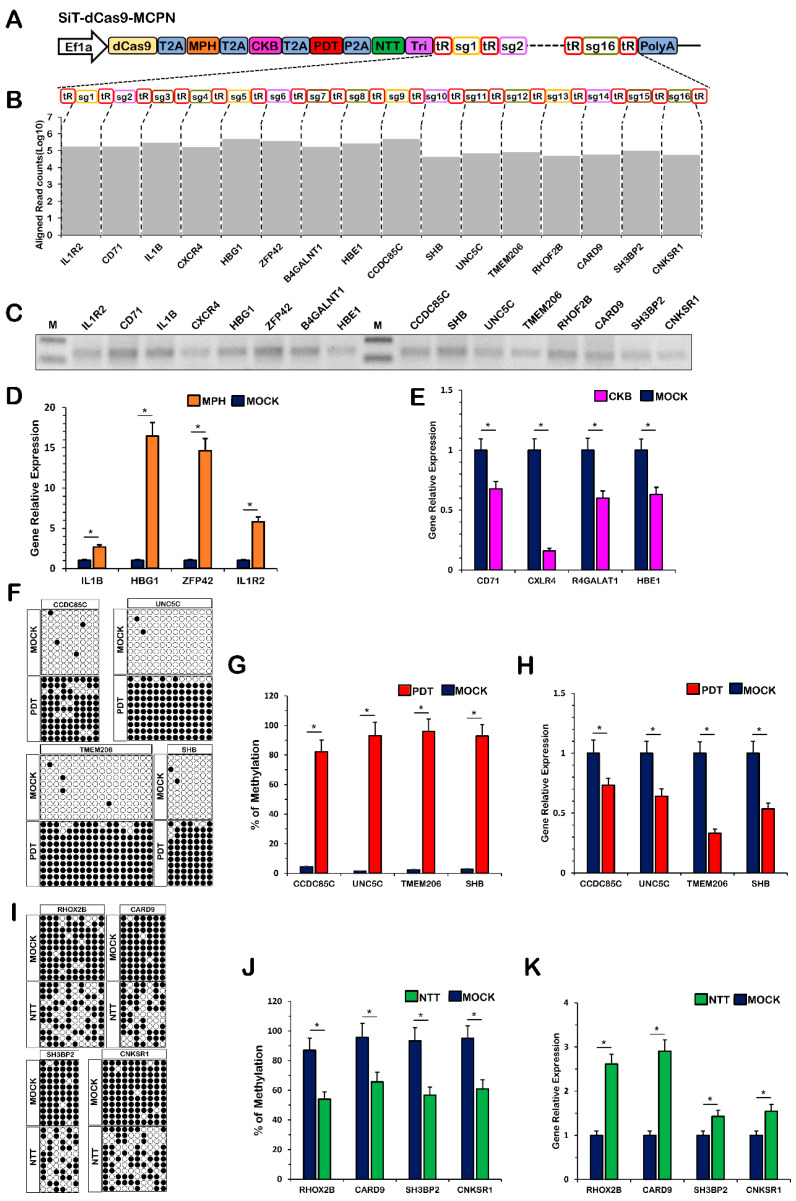
Simultaneous multiplexed orthogonal gene modulation with the CRISPR-MCPN system. (**A**) Schematic representation of the CRISPR-MCPN vector. (**B**) Quantification of mature gRNAs in cells expressing a large CRISPR array (16 gRNAs) using CRISPR-MCPN. (**C**) Quantification of mature gRNAs in cells expressing a large CRISPR array (16 gRNAs) using CRISPR. (**D**) Quantification of relative RNA expression for the *IL1B*, *HBG1*, *ZFP42* and *IL1R2* genes after multiplexed expression of 4 distinct gRNAs from the MCPN system. (**E**) Quantification of relative RNA expression for the *CD71*, *CXLR4*, *R4GALAT1* and *HBE1* genes after multiplexed expression of 4 distinct gRNAs from the MCPN system. (**F**) Methylation levels of each individual CpG in the *CCDC85C*, *SHB*, *UN5C* and *TMEM206* promoter regions. (**G**) Methylation levels of each individual CpG in the *CCDC85C*, *SHB*, *UN5C* and *TMEM206* promoter regions. (**H**) Quantification of relative RNA expression for the *CCDC85C*, *SHB*, *UN5C* and *TMEM206* genes after multiplexed expression of 4 distinct gRNAs from the MCPN system. (**I**) Methylation levels of each individual CpG in the *RHOF2B*, *CARD9*, *SH3BP2* and *CNKSR1* promoter regions. (**J**) Methylation levels of each individual CpG in the *RHOF2B*, *CARD9*, *SH3BP2* and *CNKSR1* promoter regions. (**K**) Quantification of relative RNA expression for the *RHOF2B*, *CARD9*, *SH3BP2* and *CNKSR1* genes after multiplexed expression of 4 distinct gRNAs from the MCPN system. MOCK, untransfected cells; MPH, fusion of MCP with P65 and HSF1; CKB, fusion of Com with KRAB; PDT, fusion of PCP with Dnmt3a; NTT, fusion of N22p with TET1; T2A, 2A self-cleaving peptide derived from Thosea asigna virus; P2A, 2A self-cleaving peptide derived from porcine teschovirus-1; Tri, RNA triple helix structure. Values represent the mean ± s.e.m., *n* = 3 independent experiments. * *p* < 0.05.

To explore the potential of multiplexed genome editing in the CRISPR/dCas9 context, we combined the CRISPR-MCPN system with a tRNA-gRNA array harboring 16 gRNAs targeting 16 distinct genome loci (Figure 5A). Each functional protein corresponds to four functional genome loci. cRT-PCR analysis and deep sequencing were used to confirm the generation of all 16 mature gRNA scaffolds (Figure 5B,C). The results showed that the expression of the *IL1B*, *HBG1*, *ZFP42* and *IL1R2* genes was activated by MPH (Figure 5D). The expression of the *CD71*, *CXLR4*, *R4GALAT1* and *HBE1* genes was repressed by CKB (Figure 5E). The CGC islands of the *CCDC85C*, *SHB*, *UN5C* and *TMEM206* genes promoters were methylated by PDT (Figure 5F,G), and the expression of *CCDC85C*, *SHB*, *UN5C* and *TMEM206* was downregulated (Figure 5H). The CGC islands of the *RHOF2B*, *CARD9*, *SH3BP2* and *CNKSR1* gene promoters were demethylated by NTT (Figure 5I,J), and the expression of the *RHOF2B*, *CARD9*, *SH3BP2* and *CNKSR1* genes was upregulated (Figure 5K). Overall, these data indicate that the MCPN platform enables 16 genome loci and 4 multigenome functions for gene activation, gene repression, genome methylation and genome demethylation simultaneously.

## 3. Discussion

Here, we describe an MCPN system based on CRISPR/dCas9 that can regulate multiple genome loci and multiple functions, including gene activation, gene inhibition, genome methylation and genome demethylation, simultaneously. In this system, all dcas9, functional proteins and gRNAs scaffolds are expressed under one pol II promoter. A single-transcript CRISPR/Cas9 system enables highly efficient and tunable genomic manipulations in cell types that are difficult to transfect, such as primary cells and progenitor cells. The MCPN system is useful for both basic science and therapeutic applications by enabling diverse and tunable genomic manipulations.

The flexibility and specificity of the CRISPR/Cas system has greatly increased the speed and efficiency of multiplex genome modification techniques. Based on the CRISPR/Cas system, multiplex genome editing using multiple gRNAs could be performed simultaneously at several genome sites with one type of editing event or multiple editing events [22,23,24,25,26]. Cas9 combining with the truncated sgRNA with a length of 14 bp can recognize genomic sites without cleavage activity. Cas12a binding to the truncated crRNA with a length of 15bp can recognize sites without cleavage activity. By using the truncated gRNA or crRNA, Cas9 or Cas12a has been engineered as a dual-functional genome editing system to modulate genes and cleave different targets simultaneously [22,25,27]. Other multifunctional genome editing could also be achieved by the scaffold RNA (scRNA) strategy, where RNA-hairpin sequences (i.e., MS2) were fused to the gRNA scaffold to recruit transcriptional regulators to dCas9 via aptamer–RNA-binding protein interactions. The specific interaction between aptamers and RNA-binding proteins has been used to perform dual-function systems [7], label multiplexed genomic sites [12,28] and obtain tri-functional genome editing including deaminase cytidine base editing, adenine base editing and double-strand breaks at multiple sites [14]. In this study, gRNAs fused with RNA loop MS2, PP7, com or boxB in their scaffolds were used to target genome loci of interest. These gRNAs are recruited to distinct target regions by dCas9 and recruit the effector proteins tagged to the RNA-binding viral proteins, including MPH, CKB, PDT and NTT. As a proof of principle, we created a quadruple-function CRISPR system to enable the transcriptional activation, transcription repression, DNA methylation and DNA demethylation of genomes at different individual loci simultaneously.

The CRISPR/Cas9 system is much easier and more efficient than other genome-editing tools for the editing of multiple genome loci. Many methods to express and process multiplexed gRNAs from a single transcript based on crRNA-processing mechanisms have been developed, including the use of site-specific RNA endonucleases, ribozymes and introns [15,19,29,30]. The major advantage of endogenous tRNA processing is that it can produce a large number of gRNAs by a polymerase II promoter and without any foreign auxiliary protein [17,19]. Recently, Yuan et al. (2022) reported a tRNA-gRNA system to allow the multiplexing of up to 31 gRNAs in human cells [16]. In this study, we demonstrated that 16 gRNAs could be efficiently produced from a single synthetic gene with the tRNA–gRNA architecture after the precise excision of transcripts by endogenous RNases. In this study, we stopped our testing of gRNAs with a total targeting number of 16 because of limited plasmid construction tools available for the assembly of multiple fragments with repetitive sequences (for 16 targets, 16 gRNA scaffolds and 17 tRNA^Gly^ are required). In the future this could theoretically be used to enable massively multiplexed expression of hundreds to thousands of independent gRNAs, opening up avenues for large-scale genome engineering efforts.

In most CRISPR–Cas multiplex gene modulation strategies, Cas enzymes and guide RNAs are expressed from distinct promoters or the simultaneous delivery of multiple plasmids [18,31]. In our platform, a single Pol II promoter expresses a single transcript harboring dCas9, effect proteins and a tRNA-gRNA array. The RNA triple helices from the ends of the nonpolyadenylated *MALAT1* and *MEN b* long noncoding RNAs were placed downstream of the protein ORF [20]. The triple helices additionally function to ensure transcript stability and promote efficient translation [20,22]. Consequently, by increasing dCas9 and affecting protein transcript abundance, there were enough proteins to match each gRNA. The single Pol II promoter expresses a single transcript facilitating conditional and inducible genome engineering applications in diverse cell types. This system also has the potential to simplify guide RNA delivery for modeling complex diseases caused by combinations of multidimensional gene networks where targeting multiple genes is desirable [4,32,33].

Taken together, we describe here an efficient multiplexed orthogonal genome editing strategy using dCas9 and tRNA-gRNA arrays in human cells. The scRNAs of RNA hairpins MS2, PP7, com and boxB were used for mediating transcriptional activation, transcriptional repression, DNA methylation and DNA demethylation, respectively. Therefore, in our MCPN systems, we used the tRNA-gRNA array to produce multiple gRNAs on different targets. We also took advantage of the RNA triplex sequence to realize the MCPN system expressed on a single transcript, which only requires one Pol II promoter. These findings expand the multiplex genome editing tools and provide feasible opportunities to systematically interrogate complex genetic interactions and cellular behaviors.

## 4. Materials and Methods

### 4.1. Cell Culture

The HEK293T cell line (Sigma–Aldrich, Beijing, China) was maintained in Dulbecco’s modified Eagle’s medium (DMEM) (Sigma–Aldrich, St. Louis, MO, USA) supplemented with 15% FBS (HyClone, Logan City, Utah, USA) at 37 °C with 5% CO_2_.

### 4.2. Cell Transfection

HEK293T cells were transfected using Lipofectamine 3000 (Invitrogen, Shanghai, China, #L3000001) according to the manufacturer’s instructions. Briefly, 1 × 10^6^ cells were transfected with plasmid DNA (5 μg) using Lipofectamine 3000 (Lipofectamine 3000 reagent 4 μL; P3000TM reagent 8 μL) and OPTI-MEMI (Gibco, Carlsbad, CA, USA, Cat. No. 31985-070) when about 60% of cells were fused. Samples were collected 48 h posttransfection and an additional 72 h after puro selection (1 mg/mL, Sigma, Beijing, China, #540222).

### 4.3. Plasmid Construction

The backbone plasmid used for generating CRISPR-MCPN vectors in this study was pcDNA3.1(+). CRISPR-MCPN vector was constructed by restriction enzyme-based cloning to join five DNA fragments together. Four of the five DNA fragments were synthesized commercially (GenScript, Nanjing, China). First, the DNA fragment contained Tet1 catalytic domain flanked by *Nhe*I and *Age*I restriction sites at the 5′ end of the DNA fragment. Second, the DNA fragment containing KRAB domain, PP7-binding protein, DNMT3A catalytic domain and boxB-binding protein sequences was integrated by *Nhe*I and *Age*I restriction sites. Third, the DNA fragment containing MS2-binding protein, p65 domain, HSF1 domain, Com-binding protein and part of KRAB was integrated by *Nhe*I and *BLP*I restriction sites. Fourth, the DNA fragment containing the tRNA-gRNA array (4× gRNAs) was integrated by *Bam*HI and *Psp*XI restriction sites. Finally, the EF1a promoter and dCas9 from *Streptococcus pyogenes* were integrated by *Spe*I and *Nhe*I restriction sites. CRISPR-MCPN constructs for tRNA-gRNA array (16× gRNAs) were generated by replacing tRNA-gRNA array (4× gRNAs) with tRNA-gRNA array (16× gRNAs) by *Bam*HI and *Psp*XI restriction sites.

SiT-dCas9-[GT] constructs for EGFP detection with the triplex sequence were generated by Golden Gate Cloning via *Bam*HI using the EGFP fragment. Then, the SiT-dCas9-[G] constructs for EGFP detection without the triplex sequence were generated by removing the triplex sequence using a *Pac*I restriction site. The CRISPR active vector constructs for specific binding detection were generated by replacing the gRNA-tRNA sequences with active tRNA-gRNA (4× gRNAs) sequences. Based on this plasmid, the other corresponding elements were replaced with MCP-VP65-HSF, PP7-VP65-HSF, COM-VP65-HSF, N22-VP65-HSF and DNA fragments containing MCP-HSF-VP65, PP7-HSF-VP65, COM-HSF-VP65 and N22-HSF-VP65, respectively. DNA and spacer target sequences are listed in Supplementary Table S1.

### 4.4. Quantification of mRNA Expression

Transfected HEK293T cells were harvested with trypsin, washed with Dulbecco’s phosphate-buffered saline (DPBS) and centrifuged at 300× *g* for 3 min, and then RNA was extracted using TRIzol Reagent (Thermo Fisher, Shanghai, China, #15596026). DNA was removed from RNA using an RNA Clean up Kit (OMEGA, Shanghai, China, #R6247). A cDNA library was made using a High Capacity cDNA Reverse Transcription Kit (Thermo Fisher, Shanghai, China, #4368814) supplemented with poly d(T) and random hexamer reverse transcription primer mix with 1 μg of RNA. Quantitative PCR (qPCR) was performed using TB Green™ Premix Ex Taq™ (TAKARA, Beijing, China, #RR420A) according to the manufacturer’s protocol. Primers used are indicated in Supplementary Table S2. Activation was analyzed with the Applied Biosystems 7500 instrument with the following program: 95 °C for 3 min, followed by 40 cycles at 95 °C for 10 s, 63 °C for 20 s and then 72 °C for 5 s. Gene expression levels were normalized to the geometric mean of the control gene with ΔΔCt calculated by comparison to the untransfected samples.

### 4.5. Western Blot

HEK293T cells were seeded at 80% confluency and transfected with sterilized plasmids to 5 μg, according to the operation in Section 4.2. Samples were collected 48 h posttransfection and an additional 72 h after puro selection (2 μg/mL, Sigma, #A1113803, Beijing, China). Total cellular protein was extracted from HEK293T cells by lysing cells in ice-cold radioimmunoprecipitation assay buffer containing protease inhibitors, 50 mM Tris–HCl, pH 8.0, 150 mM NaCl, 1% NP-40, at 4 °C and phosphatase inhibitors (R&D Systems, Shanghai, China # 5140). After centrifugation at 12,000× *g* (30 min at 4 °C), the supernatants were collected, and protein concentrations were measured using the bicinchoninic acid (BCA) Protein Assay kit (Beyotime, Beijing, China #P0012S). Samples containing 50 μg of protein were mixed with loading dye and subjected to sodium dodecyl sulfate–polyacrylamide gel electrophoresis (SDS–PAGE) using 10% polyacrylamide gels (30% acrylamide-bisacrylamide, 1.5 M Tris–HCl, pH 8.8, 10% SDS, 10% ammonium persulfate and TEMED) (Beyotime, Beijing, China #ST728) and transferred onto polyvinylidene difluoride membranes (Millipore, MA, USA #03010040001). Membranes were blocked in 5% Tris-buffered saline (10 mM Tris-HCl, pH 8.0, 150 mM NaCl, 0.5% Tween-20 and 5% fat-free dry milk) for 1 h at room temperature. The membranes were then probed overnight at 4 °C with specific primary antibodies against Myc-Tag (19C2) (Abmart, Shanghai, China, A#M20002), HA-Tag (19C2) (Abmart, Shanghai, China, #M20003), His-Tag (19C2) (Abmart, Shanghai, China, #M20001), Flag-Tag (Genscript, Nanjing, China, #A00170), Cas9 (Genscript, Nanjing, China, #A01885) and ACTB (1:1000) (Sigma, #A1978, Shenyang, China), which acted as a loading control. The following day, the membranes were washed and incubated with secondary antibody, a horseradish peroxidase (HRP)-conjugated anti-rabbit goat IgG antibody (1:1000) (Sigma, #A9169, Beijing, China). Membranes were developed using an enhanced chemiluminescence (ECL) kit (Thermo Fisher Scientific, #34577 Shanghai, China) according to the manufacturer’s instructions. Band intensity was quantified by densitometry using ImageJ software (version 1.38). The protein ladder (Thermo, Shanghai, China, #26634) was used to measure the molecular weight of proteins. The relative level of protein expression was expressed as the density ratio of the protein compared to ACTB levels in the same sample.

### 4.6. Immunocytochemistry

Cells were washed in PBS and fixed in 4% paraformaldehyde for 30 min. After 3 washes in DPBS, cells were permeabilized with 0.3% Triton-X-100 for 60 min, blocked with PBS containing 2% BSA for 60 min and incubated with primary antibodies overnight at 4 °C. Then, the cells were treated with secondary antibody for 60 min at room temperature and mounted with Hoechst 33,342 (Sigma, Beijing, China) after 3 washes in DPBS.

The following antibodies were used in this study: anti-Myc-Tag (Abmart, Shanghai, China, A#M20002), anti-HA-Tag (Abmart, Shanghai, China, #M20003), anti-His-Tag (Abmart, Shanghai, China, #M20001), anti-Flag-Tag (Genscript, Nanjing, China, #A00170) and anti-dCas9 (Genscript, Nanjing, China, #A01885). Goat anti-rabbit secondary antibody (1:5000) (Santa Cruz, Dallas, TX, USA, #sc-2004). Samples were assessed by fluorescence microscopy at 530 nm and 480 nm excitation wavelengths (Olympus BX51, Olympus, Tokyo, Japan).

### 4.7. Mature gRNA Quantification

Total RNA was extracted from protoplasts using TRIzol Reagent (Life Technologies, Shanghai, China) according to the manufacturer’s instructions. One microgram of total RNA was reverse-transcribed to produce cDNA using the specific primer gRNA-R along with MuMLV (New England Biolabs, Beijing, China) according to the manufacturer’s instructions. Then, PCR was performed with the following 50 µL reaction: one twentieth of 1st cDNA, 1 × buffer, 1 × higher GC buffer, 0.2 mM dNTPs, 0.5 µM forward primers, 0.5 µM reverse primers and 1 U of DNA polymerase (NEBNext Q5, #M0543, Beijing, China), with the following conditions: 98 °C for 5 min, followed by 33 three-step cycles at 95 °C for 10 s, 53 °C for 10 s and 72 °C for 12 s. The resulting PCR products were analyzed with 1% agarose gel electrophoresis. The purified cRT-PCR products were delivered to Annoroad Gene Technology for deep sequencing (Yiwu, Zhejiang, China). See Supplementary Table S3 for primer sequences used in cRT–PCR.

### 4.8. Targeted BS-PCR Primer Design and Sequencing

Bisulfite modification was performed on 0.3 µg of DNA from each sample using the EZ DNA Methylation-Gold Kit (Zymo Research, Beijing, China) according to the instruction manual. PCR primers to amplify the promoters were designed with Methyl Primer Express Software v1.0, which was also used to predict CpG islands and CpG sites in the sequences. See Supplementary Table S4 for primer sequences used in BS-PCR. The amplification of bisulfite-modified DNA was performed using Hot Start EpiMark TaqTM polymerase (NEB, Beijing, China, #M0490), one twentieth of 100 ng DNA, 1 × EpiMark Reaction buffer, 0.5 µM forward primers, 0.5 µM reverse primers and Hot Start EpiMark TaqTM polymerase (NEB, Beijing, China, #M0490) with the following conditions: 98 °C for 5 min, followed by 33 three-step cycles at 95 °C for 30 s, 53 °C for 30 s, 68 °C for 24 s and 68 °C for 5 min. The PCR products were separated on 1% agarose gels and purified, followed by sequencing (Invitrogen, Beijing, China). The presence of a cytosine residue after bisulfite treatment shows that the cytosine residue was protected from bisulfite modification by methylation. Methylated and nonmethylated CpG dinucleotides of each clone are illustrated with closed and open circles, respectively. At least ten clones were sequenced and analyzed for each sample.

### 4.9. Statistics

Experimental data are expressed as the means ± SEMs. All statistical analyses were performed with SPSS 19 software (Somers). Student’s *t* test with Bonferroni correction was used to evaluate statistical significance between the different treatment groups. Unless otherwise noted, experiments in this study were performed using three independent biological replicates. *p* < 0.05 indicates significant differences.

## Data Availability

The authors declare that all data supporting the findings of this study are available within the paper and its supplemental information files.

## Supplementary Materials

The supporting information can be downloaded at https://www.mdpi.com/article/10.3390/ijms24108535/s1.
