# Supplementary material for: Multiplexed Gene Engineering Based on dCas9 and gRNA-tRNA Array Encoded on Single Transcript
Source: Int J Mol Sci. 2023 May 10;24(10):8535. doi: 10.3390/ijms24108535 (PMC10218229; doi:10.3390/ijms24108535)
Supplement: Supplementary file 1 [file ijms-24-08535-s001.zip › Supplementary Table S1. sgRNA Target Sites.pdf]

Supplementary Table S1. sgRNA Target Sites

Gene Name	sgRNA sequence (5'-3')	PAM Sequence	
IL1B	AAAAACAGCGAGGGAGAAAC	TGG	Gene Activation
HBG1	GGCTAGGGATGAAGAATAAA	AGG	
ZFP42	GGGTCTTGGGAGGGGGCGCA	GGG	
IL1R2	GACCCAGCACTGCAGCCTGG	GGG	
CD71	GGACGCGCTAGTGTGAGTGC	GGG	Gene Repression
CXCR4	GCAGGTAGCAAAGTGACGCCGA	GGG	
B4GALNT1	GCCGAAGCAGCCGCAACGAGC	CGG	
HBE1	GCTAGTGATTGCAGCTGTGT	CGG	
CCDC85C-CTCF	TACAAAGGATGGAACGCGGC	AGG	DNA Methylation
SHB-CTCF	CTGTGTGCTAAACCTCCCGT	TGG	
UNC5C	GTCCAGACTTCGGCGTGCGG	CGG	
TMEM206	GTGCTGCGTCCGTGCGCCG	CGG	
RHOF2B	GCTTGGCCTTGCCCGGATGA	GGG	DNA Demethylation
CARD9	TGGGAGCAGCTTTCCTCTGG	AGG	
SH3BP2	TGAGGTCCTGAAAGCTGCCT	GGG	
CNKSR1	TGTGAGCCCAGGTATGCAGT	AGG	
