# Supplementary material for: Multiplexed Gene Engineering Based on dCas9 and gRNA-tRNA Array Encoded on Single Transcript
Source: Int J Mol Sci. 2023 May 10;24(10):8535. doi: 10.3390/ijms24108535 (PMC10218229; doi:10.3390/ijms24108535)
Supplement: Supplementary file 1 [file ijms-24-08535-s001.zip › Supplementary Table S2. Primer Sequence for RT-PCR.pdf]

Supplementary Table S2. Primer Sequence for RT-PCR

Primer Name	Primer sequence(5'-3')	Analysis
CXCR4-1	GAAGCTGTTGGCTGAAAAGG	CXCR4 expression analysis
CXCR4-2	CTCACTGACGTTGGCAAAGA	
CD71-1	AAAATCCGGTGTAGGCACAG	CD71 expression analysis
CD71-2	GCACTCCAACTGGCAAAGAT	
B4GALNT1-1	AGAGGGTCAGGCAGATCTCA	B4GALNT1 expression analysis
B4GALNT1-2	TGCTGTGTTGGTCTGGTAGC	
HBE1-1	TCACTAGCAAGCTCTCAGGC	HBE1 expression analysis
HBE1-2	AACAACGAGGAGTCTGCCC	
IL1B-1	AAACAGATGAAGTGCTCCTTCC	IL1B expression analysis
IL1B-2	AAGATGAAGGGAAAGAAGGTGC	
IL1R2-1	ATGTTGCGCTTGTACGTGTTG	IL1R2 expression analysis
IL1R2-2	CCCGCTTGTAATGCCTCCC	
ZFP42-1	AGAAACGGGCAAAGACAAGAC	ZFP42 expression analysis
ZFP42-2	GCTGACAGGTTCTATTTCCGC	
HBG1-1	GCTGAGTGAACTGCACTGTGA	HBG1 expression analysis
HBG1-2	GAATTCTTTGCCGAAATGGA	
RHOXF2B-1	GGCAAGAAGCATGAATGTGA	RHOXF2B expression analysis
RHOXF2B-2	TGTCTCCTCCATTTGGCTCT	
CARD9-1	CAGGCTCCTGGTGTGTCTG	CARD9 expression analysis
CARD9-2	CTCCAGCACTCGTCATCGT	
SH3BP2-1	ATGTGTTGGGTCAGCACCA	SH3BP2 expression analysis
SH3BP2-2	CAGGCATGGTTAGCAGGTTC	
CNKSR1-1	GGCAAAACAGGAGCTGATTC	CNKSR1 expression analysis
CNKSR1-2	TAGTCCTGCAGGGAGTCGTC	
UNC5C-1	AGCCCAACTGGCGTAATCCT	UNC5C expression analysis
UNC5C-2	ACACCACCGTTTCATGTCTTCC	
SHB-1	TCCACTACTACACCACCAGAAAGC	SHB expression analysis
SHB-2	CACAGGGTCCTCACAGCCAC	
TMEM206-1	CTCCATCGTGCCGCCTCATT	TMEM206 expression analysis
TMEM206-2	CGTCTCCTTGTCCTGCTCGTCT	
CCDC85C-1	CTGTGAGGAGGACCTGAGTGAGAA	CCDC85C expression analysis
CCDC85C-2	GCCGTATGGAGGGCTTGGA	
ACTB sense	GATGCAGAAGGAGATCACTGC	Normalization for expression in HEK293
ACTB antisense	GTACTTGCGCTCAGGAGGAG	
