# Supplementary material for: Multiplexed Gene Engineering Based on dCas9 and gRNA-tRNA Array Encoded on Single Transcript
Source: Int J Mol Sci. 2023 May 10;24(10):8535. doi: 10.3390/ijms24108535 (PMC10218229; doi:10.3390/ijms24108535)
Supplement: Supplementary file 1 [file ijms-24-08535-s001.zip › Supplementary Table S3. Primer Sequence for cRT-qPCR.pdf]

Supplementary Table S3. Primer Sequence for cRT-qPCR

Primer Name	Primer sequence (5'-3')	Analysis
IL1B-S	AAAAACAGCGAGGGAGAAACGTTTTAGAGCTAGGCCAACA	IL1B sgRNA expression analysis
CCDC85C-S	TACAAAGGATGGAACGCGGCGTTTTAGAGCTAGGAGCAGA	CCDC85C sgRNA expression analysis
CD71-S	GGACGCGCTAGTGTGAGTGCGTTTTAGAGCTAGGCCACTG	CD71 sgRNA expression analysis
ROHX2B-S	GCTTGGCCTTGCCCGGATGAGTTTGAGAGCTAGGGCCCTG	ROHX2B sgRNA expression analysis
HBG1-S	GGCTAGGGATGAAGAATAAAGTTTTAGAGCTAGGCCAACA	HBG1 sgRNA expression analysis
SHB-S	CTGTGTGCTAAACCTCCCGTGTTTTAGAGCTAGGAGCAGA	SHB sgRNA expression analysis
CXCR4-S	GCAGGTAGCAAAGTGACGCCGAGTTTTAGAGCTAGGCCAC	CXCR4 sgRNA expression analysis
CARD9-S	TGGGAGCAGCTTTCCTCTGGGTTTGAGAGCTAGGGCCCTG	CARD9 sgRNA expression analysis
ZFP42-S	GGGTCTTGGGAGGGGGCGCAGTTTTAGAGCTAGGCCAACA	ZFP42 sgRNA expression analysis
UNC5C-S	GTCCAGACTTCGGCGTGCGGGTTTTAGAGCTAGGAGCAGA	UNC5C sgRNA expression analysis
B4GALTN1-S	GCCGAAGCAGCCGCAACGAGCGTTTTAGAGCTAGGCCACT	B4GALTN1 sgRNA expression analysis
SH3BP2-S	TGAGGTCCTGAAAGCTGCCTGTTTGAGAGCTAGGGCCCTG	SH3BP2 sgRNA expression analysis
IL1R2-S	GACCCAGCACTGCAGCCTGGGTTTTAGAGCTAGGCCAACA	IL1R2 sgRNA expression analysis
TMEM206-S	GTGCTGCGTCCGTGCGCCGGTTTTAGAGCTAGGAGCAGAC	TMEM206 sgRNA expression analysis
HBE1-S	GCTAGTGATTGCAGCTGTGTGTTTTAGAGCTAGGCCACTG	HBE1 sgRNA expression analysis
CNKSR1-S	TGTGAGCCCAGGTATGCAGTGTTTGAGAGCTAGGGCCCTG	CNKSR1 sgRNA expression analysis
cRT-PCR-A	GCACCGACTCGGTGC	Reverse primer
