# Supplementary material for: Multiplexed Gene Engineering Based on dCas9 and gRNA-tRNA Array Encoded on Single Transcript
Source: Int J Mol Sci. 2023 May 10;24(10):8535. doi: 10.3390/ijms24108535 (PMC10218229; doi:10.3390/ijms24108535)
Supplement: Supplementary file 1 [file ijms-24-08535-s001.zip › Supplementary Table S4.pdf]

Supplementary Table S4. Primers for targeted bisulfite sequencing

Primer Name	Primer Sequence(5'-3')	Primer Target
RHOXF2B-B1	GTTATAAAATGGGTTTGTATAATTTAGTAT	RHOXF2B promoter
RHOXF2B-B2	AAAACCTCCTCTCTTACTTTTCTACTTC	
CARD9-B3	GGTTATTAGGGATTGTTTTTTTGTG	CARD9 promoter
CARD9-B4	ATCTTCCAAAAACCACCTACACTAC	
SH3BP2-B1	TTATAGGGTAGAAGGTAGGAAGTGT	SH3BP2 promoter
SH3BP2-B2	ATCTCCCAAACATATAAAACCTAAC	
CNKSR1-B1	TTTTTTTAGGTTTGGGTTTTGG	CNKSR1 promoter
CNKSR1-B2	AATAACCCACCCACCTTAACTTC	
UNC5C_promoter_1 Forward	TTTTTGTTTTATTGTAAAGGGGTTATA	UNC5C promoter_1
UNC5C_promoter_1 Reverse	CCAAAAAAACCAACTATAAATTTACCC	
UNC5C_promoter_2 Forward	CTCAATAAAAATCCTACTACCCCAAT	UNC5C promoter_2
UNC5C_promoter_2 Reverse	TTTAATTTATTTTTTGAGATGGGTA	
UNC5C_promoter_3 Forward	GGTTTAGTGTGTTTAGAGATTTAGTGAAGA	UNC5C promoter_3
UNC5C_promoter_3 Reverse	TTTCCCAACTAATCAAAAAACTTTAATTAT	
UNC5C_promoter_4 Forward	GTTATTTTTTAGAGGAGTTGTGTTG	UNC5C promoter_4
UNC5C_promoter_4 Reverse	TCCACTTTCTCCAAAAACAAAAACC	
UNC5C_promoter_5 Forward	TAAAGTTTTTTGATTAGTTGGGAAAGT	UNC5C promoter_5
UNC5C_promoter_5 Reverse	CCTTTTAACCCCTACCTTTAAAAAA	
UNC5C_promoter_6 Forward	TTGAGTAGGGTTAGGGTAGGTAGTA	UNC5C promoter_6
UNC5C_promoter_6 Reverse	AAAAAAAACCTTCAAACTCCTCCT	
UNC5C_promoter_7 Forward	TTTAAAAATCCCTCTTTCCCAATAC	UNC5C promoter_7
UNC5C_promoter_7 Reverse	TTTTAGGATGTGAATTTTTTTGGTTAT	
UNC5C_promoter_8 Forward	GTTTTATGTAAGTAAGGATTGTAGGGAGAT	UNC5C promoter_8
UNC5C_promoter_8 Reverse	CCCCAACCAAAAAAAACTTCAATTC	
UNC5C_promoter_9 Forward	TGGGATTGGGATATTTGTTGTAAATGT	UNC5C promoter_9
UNC5C_promoter_9 Reverse	TCCTACTACCCCAATCTTAACACAAC	
UNC5C_promoter_10 Forward	TAAAGGTAGGGGTTAAAAGGGGGAG	UNC5C promoter_10
UNC5C_promoter_10 Reverse	TAAACCTCCTTAACCACCCAACAAC	
SHB promoter_1 Forward	TTTTAGTGTTGGGTATTTTGGGTTT	SHB promoter_1
SHB promoter_1 Reverse	ATCCCCCTTTATCTACCTAATAAATAACAA	
SHB promoter_2 Forward	TTTAGGGGAAGGGAAAATTTTTTTT	SHB promoter_2
SHB promoter_2 Reverse	AACTCAAAACCTAAACCACAACCAC	
CCDC85C promoter_1 Forward	AAGGGTAGAGGGGGATTTTTTGTT	CCDC85C promoter_1
CCDC85C promoter_1 Reverse	AAACCAATTTCCCAAATCTCCTACC	
CCDC85C promoter_2 Forward	AAGTTGTTTGTGTTTGTGGGTTTAGG	CCDC85C promoter_2
CCDC85C promoter_2 Reverse	ACCAAACATCCTCCCTCCTACTTCT	
TMEM206 promoter_1 Forward	GTATTTTTTGGTAGGATGTGGAG	TMEM206 promoter_1
TMEM206 promoter_1 Reverse	ACTAACCCAAAATCACATACAAACCTC	
TMEM206 promoter_2 Forward	TTTTAATAAAGAGAGGAAGTGAGAGA	TMEM206 promoter_2
TMEM206 promoter_2 Reverse	AATCATAACACTAACCAAAACTTC	
